# Emergency Critical Care as a Continuum: The All India Institute of Medical Sciences (AIIMS) Three-Tiered Framework for the Assessment and Management of Critically Ill Patients in the Emergency Department

**DOI:** 10.7759/cureus.115468

**Published:** 2026-08-30

**Authors:** Takshak Shankar, Parvathy Sasidharan, Nidhi Kaeley, Ratender K Singh, Ashima Sharma, Amrutha Priya CP, Jitendra K Yadav, Devinder K Lalotra, Akshaya Balagopal

**Affiliations:** 1 Emergency Medicine, All India Institute of Medical Sciences Rishikesh, Rishikesh, IND; 2 Emergency Medicine, Dr. Moopen's Medical College, Wayanad, IND; 3 Emergency Medicine, Sanjay Gandhi Post Graduate Institute of Medical Sciences, Lucknow, IND; 4 Emergency Medicine, Nizam's Institute of Medical Sciences, Hyderabad, IND

**Keywords:** critical emergency medicine, emergency critical care, emergency medicine training, longitudinal emergency critical care, resuscitation

## Abstract

Emergency critical care (ECC) is increasingly recognized as an indispensable component of the critical care continuum, beginning at the point of first medical contact in the emergency department (ED). As critically ill patients spend longer periods in the ED and emergency physicians assume an expanding role in providing ongoing critical care, existing assessment frameworks may not fully address the evolving cognitive demands of ECC. This article describes a conceptual three-tier framework intended to provide a pragmatic strategy for the assessment and management of critically ill patients throughout their ED course. The framework was conceived and implemented at All India Institute of Medical Sciences, Rishikesh. The framework aligns patient assessment and management with three complementary clinical priorities. Tier 1 emphasizes physiological rescue through simultaneous assessment and stabilization using an expanded ABCDEF approach, with a focus on identifying and managing immediately life-threatening and time-sensitive conditions. Following initial stabilization, Tier 2 focuses on diagnostic reasoning, integrating clinical evaluation to establish an ED working diagnosis and guide subsequent management. Tier 3 addresses longitudinal ECC through structured reassessment, optimization of organ support, diagnostic refinement, and prevention of secondary complications while patients remain under ED care. Although presented as three sequential tiers for conceptual clarity, the framework is intended to be dynamic and iterative, allowing movement between tiers according to the patient's evolving clinical status. By integrating principles of emergency medicine and critical care, this conceptual framework provides a cognitive scaffold for clinicians managing critically ill patients in the ED and may support continuity of care, education, and quality improvement. As the framework is currently conceptual, further studies are required to evaluate its feasibility and potential impact on patient outcomes.

## Introduction

Critical illness refers to a potentially reversible state of severe physiological derangement, characterized by instability of vital functions and/or dysfunction of vital organs, placing patients at substantial risk of imminent mortality in the absence of timely intervention [1]. Although the underlying etiologies and clinical trajectories of critical illness may vary considerably, all critically ill patients share a common requirement for early recognition and prompt initiation of appropriate interventions. For most patients presenting with acute medical, surgical, or traumatic conditions, the emergency department (ED) represents the first point of hospital-based care and therefore plays a pivotal role in the early identification and initial management of critical illness [2].

A European group of emergency and critical care physicians has recently highlighted the imperative for timely ED-based critical care delivery, emphasizing that the ED management phase constitutes an integral component of the continuum of care for critically ill patients [3]. Contemporary EDs routinely provide advanced hemodynamic monitoring, targeted organ support, and complex clinical decision-making pending definitive disposition of critically ill patients, with emergency physicians (EPs) increasingly engaged in the delivery of complex and high-acuity care [4-6]. Consequently, critically ill patients may be considered to enter the continuum of critical care at the time of first medical contact in the ED rather than only upon physical admission to an ICU [2,4].

The ED, however, functions within a singularly challenging clinical environment characterized by diagnostic uncertainty, time constraints, limited resources, frequent interruptions, and complex patient-physician interactions [7-9]. Within this setting, EPs must simultaneously prioritize physiological stabilization, recognition of time-sensitive conditions, diagnostic reasoning, and disposition planning while making critical decisions under conditions of uncertainty [10,11]. Despite growing recognition that critical care begins well before ICU admission, a structured framework to guide the assessment and management of critically ill patients across the continuum of emergency critical care (ECC) remains lacking.

## Technical report

The AIIMS three-tiered model for assessment of critically ill patients in the ED

To operationalize ECC as a continuum beginning at the point of first medical contact, we describe the AIIMS Three-Tiered Framework for the assessment and management of critically ill patients in the ED. The framework was conceptualized and developed at our parent institution, the All India Institute of Medical Sciences (AIIMS), Rishikesh. It integrates three interrelated yet distinct domains of ECC: physiological rescue, diagnostic reasoning, and longitudinal critical care management. Importantly, it is intended to guide the care of all patients requiring ECC, irrespective of their eventual disposition, and is therefore applicable to both patients who require subsequent ICU admission and those whose critical illness resolves during the ED phase. The conceptual basis of this model is summarized in Table 1.

**Table 1:** Conceptual basis of the three-tier framework for emergency critical care

Tier	Predominant Clinical Focus	Dominant Cognitive Task	Principal Objective
Tier 1	What physiological derangements require immediate intervention?	Physiological rescue	Stabilization and identification of time-sensitive conditions
Tier 2	What pathophysiological process explains the patient's presentation?	Diagnostic reasoning	Establishment of an ED working diagnosis and management plan
Tier 3	What interventions are required to optimize ongoing critical illness management?	Longitudinal critical care management	Ongoing organ support, reassessment, and prevention of secondary injury

The first tier focuses on the physiological stabilization of the patients, which can be approached through an ABCDEF approach, an expansion of the conventional ABCDE (Airway, Breathing, Circulation, Disability, and Exposure) framework [12,13]. It may be modified to x-ABCDEF (x: Exsanguinating hemorrhage) approach for trauma patients [14]. The availability of greater diagnostic and therapeutic resources in the ED permits a more detailed evaluation of each component. In this tier, assessment and treatment occur simultaneously, continuously, and expeditiously. As many life-saving interventions require time for their clinical effects to become evident [15], this interval may be utilized to obtain a focused history, perform a focused physical examination, and pursue targeted investigations, represented by the additional “F” component of the ABCDEF approach. Guided by the presenting complaint, this focused assessment aims to identify time-sensitive conditions where delays in treatment may adversely affect outcomes [16] and to inform appropriate therapeutic decisions. Establishing a definitive diagnosis is not the primary objective at this stage. A standardization of this approach may improve patient outcomes and reduce ambiguity in clinical decision-making [17]. A brief description of the tier 1 approach is provided in Table 2.

**Table 2:** Tier 1: The ABCDEF approach to the initial assessment of critically ill non-trauma patients

Parameter	Assessment
Airway	Assessment of airway status, including identification of a patent, threatened, or obstructed airway, with initiation of appropriate management.
Breathing	Assessment of respiratory parameters, including respiratory rate, SpO₂, work of breathing, use of accessory muscles, cyanosis, chest deformities, tracheal position (when clinically indicated), respiratory movements, subcutaneous emphysema, percussion findings (when indicated), and breath sounds, and their appropriate management.
Circulation	Assessment of cardiovascular status including pulse, blood pressure, jugular venous distension, capillary refill time, pedal edema, and heart sounds, and their appropriate management.
Disability	Neurological assessment including level of consciousness using the Glasgow Coma Scale (GCS) or Alert, Verbal, Pain, Unresponsive (AVPU) scale, pupillary response, focal neurological deficits, signs of meningeal irritation when clinically indicated, pain assessment, and random blood sugar, and its appropriate management.
Exposure	Assessment of temperature, skin findings including rash, and injuries, with appropriate management based on identified abnormalities.
Focused Evaluation	The focused evaluation comprises the focused history and the focused physical examination, which may be followed by focused investigations. The objective is to rapidly characterize the acute illness, identify potential time-sensitive emergencies, and prioritize immediate management. Emphasis should be placed on features that influence urgent clinical decision-making rather than on obtaining a comprehensive chronology of the illness. (i) Focused History: The focused history should be directed toward the patient's presenting complaint and supplemented by pertinent details regarding comorbidities, prescription medications, allergies, addictions, and psychosocial factors. (ii) Focused Examination: The focused examination should be directed by the patient's presenting complaint and the clinical context. (iii) Focused Investigations: Investigations should be guided by the findings of the focused history and physical examination. These investigations are not intended for a detailed diagnostic evaluation but rather as targeted tools to support immediate management. Some examples include point-of-care ultrasound (POCUS), electrocardiography (ECG), arterial blood gas analysis (ABG), urine ketone testing, urine pregnancy testing (UPT), and neuroimaging.

The second tier follows initial physiological stabilization and the satisfactory management of immediately life-threatening and time-sensitive conditions. This stage represents the transition from physiological rescue to diagnostic reasoning and individualized management. It includes a detailed history of the presenting illness, historical review of systems, review of available medical records, and a pertinent physical examination supplemented by a review of systems. The objectives are to formulate a differential diagnosis, establish an ED working diagnosis, and develop a structured plan for further investigations, treatment, monitoring, and disposition.

The third tier represents ED critical care management, which adapts core intensive care principles to the unique needs of acutely ill patients in the ED. This phase emphasizes structured reassessment, targeted interventions, optimization of organ support, prevention of secondary complications, and facilitation of recovery. The approach may be conceptualized using an ABCDEF framework, where A represents Analgesia and sedation; B, Breathing, ventilatory management (including Spontaneous Breathing Trials and Spontaneous Awakening Trials) and Bowel care; C, Circulation (including advanced hemodynamic monitoring), Catheter care, and Complication prevention (including disease complications, infection prevention, ulcer prophylaxis, and thromboprophylaxis); D, Diagnostic refinement and Drug optimization; E, Elevation of the head of bed and Early mobilization; and F, Feeding, glycemic control, and Family integration. This framework builds upon established ICU care bundles [18] by integrating diagnostic refinement with targeted therapeutic interventions aimed at reversing the underlying disease process while optimizing supportive care throughout the patient's ED course.

This framework seeks to provide emergency medicine residents and physicians with a structured cognitive scaffold to support the delivery of ECC, spanning the continuum from first medical contact in the ED to definitive disposition, as summarized in Figure 1.

**Figure 1:**
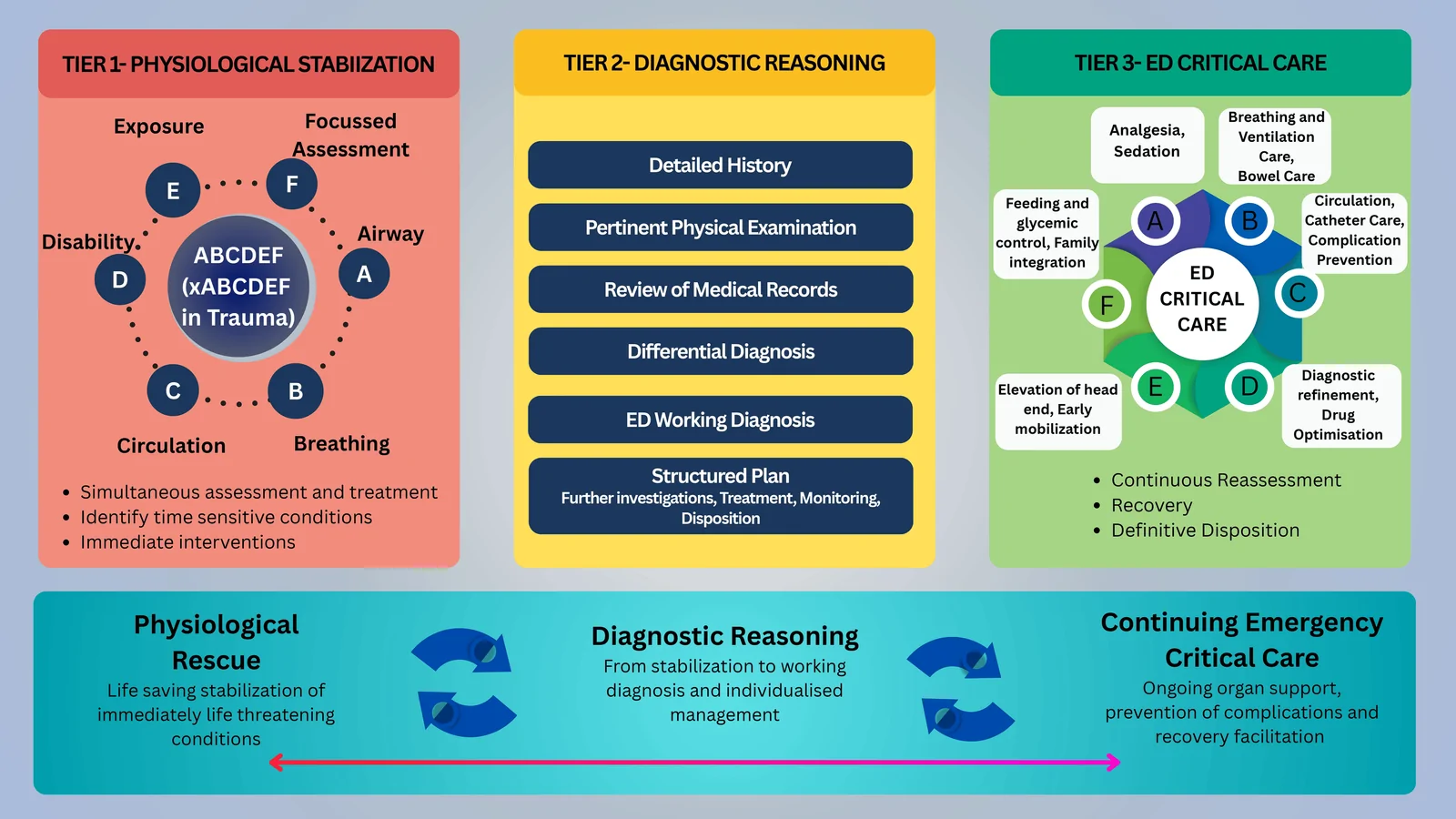
The three-tiered assessment of critically ill patients in the emergency department A dynamic and iterative framework integrating physiological stabilization, diagnostic reasoning, and emergency critical care. Image created by the authors using Canva (Canva Inc., San Francisco, CA).

## Discussion

The evolving convergence of critical care and emergency care

Historically, ED operations and training have been centered on diagnostic evaluation and initial physiological stabilization rather than the provision of sustained inpatient-level critical care. Consequently, structured approaches to the ongoing management of critically ill patients during prolonged ED stays remain relatively underdeveloped. However, this traditional paradigm has been increasingly challenged by the growing prevalence of prolonged ED boarding worldwide. Recognizing this evolving reality, the ED-Critical Care Medicine (CCM) Boarding Task Force emphasized that targeted critical care interventions can be effectively delivered to critically ill patients irrespective of their physical location or ultimate disposition. The task force further acknowledged that EPs routinely provide prolonged critical care within the ED and highlighted opportunities to optimize the delivery of such care [5].

Concurrently, accumulating evidence suggests that the timely initiation of high-quality critical care within the ED can have a substantial positive impact on the ultimate patient outcomes by facilitating rapid physiological stabilization, preventing the progression of organ dysfunction, reducing the length of hospital stay and, in selected cases, obviating the need for ICU admission altogether [19-26]. Additionally, ED-ICU models have demonstrated potential to enhance hospital fiscal performance and operational efficiency while enabling delivery of higher-value care without increasing overall healthcare costs for patients [27,28].

The principles of ECC largely overlap with those of intensive care delivered in the ICU; however, certain aspects assume greater significance during the acute phase of critical illness. ECC should therefore not be conceptualized as an extension of intensive care medicine into the ED, as may be implied by frameworks such as ICU outreach or “critical care without walls” [29,30]. Rather, ECC represents an early phase of critical care centered on the management of evolving physiological instability and trajectory-guided clinical decision-making, facilitating either early de-escalation or definitive allocation to the ICU. Beyond initial resuscitation and physiological stabilization, a fundamental component of this phase is the timely identification and treatment of the underlying pathology responsible for critical illness, as effective reversal of critical illness is contingent upon accurate diagnosis and appropriate management of the causative condition [15]. Accordingly, the diagnostic process, incorporating history, examination, and investigations, must be integrated with immediate resuscitative priorities [31].

Existing emergency medicine frameworks emphasize physiological stabilization and the recognition of life-threatening conditions but offer limited guidance for the longitudinal management of critical illness. Conversely, critical care frameworks are primarily designed for patients with established diagnoses being managed within the relatively controlled environment of the ICU [5,15,32]. Therefore, ECC requires specialized structured cognitive frameworks that preserve the foundational principles of emergency medicine while simultaneously addressing the evolving needs of critically ill patients during the acute phase of illness.

Rationale of the three-tiered framework

The ED represents one of the most cognitively demanding environments in medicine. EPs must make high-stakes decisions under conditions of diagnostic uncertainty, incomplete information, time pressure, frequent interruptions, competing clinical priorities, and challenging patient-physician interactions [7-9,33]. Consequently, the assessment and management of critically ill patients require clinicians to continuously adapt their decision-making strategies while balancing multiple competing objectives [10,11,16].

ECC represents a distinct domain that originates from emergency medicine while incorporating principles of intensive care, with a primary focus on resuscitation, early physiological stabilization, and trajectory-guided clinical decision-making rather than the provision of definitive ICU-level care. Although temporally bounded by local resources, it constitutes an indispensable component of the broader critical care continuum [4]. Thus, existing emergency medicine and critical care frameworks incompletely address the cognitive demands of ECC. The management of critically ill patients in the ED requires physicians to navigate three distinct, although overlapping, cognitive tasks. During the initial phase of care, the predominant objective is physiological rescue through the rapid identification and treatment of immediately life-threatening and time-sensitive conditions. Following stabilization, the emphasis shifts toward diagnostic reasoning, involving the generation and refinement of differential diagnoses and formulation of a structured management plan. Subsequently, during prolonged ED stays or ongoing critical illness, the focus transitions to longitudinal critical care management, encompassing diagnostic refinement, continuous reassessment, optimization of organ support, prevention of secondary complications, and facilitation of recovery [2,4]. Failure to recognize these evolving priorities may contribute to fragmented care, cognitive overload, and delays in diagnosis or treatment [16,33].

AIIMS Rishikesh serves as an apex referral center for a catchment population of approximately 15 million people and receives more than 50,000 annual ED visits involving patients requiring critical care. With hospital bed occupancy exceeding 95%, a substantial proportion of critically ill patients in the ED experience prolonged waiting times for ICU beds. In accordance with the recommendations of the ED-CCM Boarding Task Force [5], ED critical care was initiated at our institution in 2021. The department currently comprises a 30-bed resuscitation area and a 25-bed ED critical care area, including 10 ICU and 15 high-dependency unit (HDU) beds. The ICU provides advanced critical care consistent with level III capabilities as defined by national guidelines, including multiparameter monitoring, invasive and non-invasive mechanical ventilation, and infusion therapy, while the HDU provides monitored intermediate-level care. Patients remain in these areas until clinical improvement permits transfer to an appropriate level of care or discharge, as applicable. The resuscitation, ICU, and HDU areas are staffed round the clock by trained nursing personnel and emergency medicine residents under the supervision of emergency medicine consultants, with a 1:1 nurse-to-patient ratio in the ICU. Advanced point-of-care ultrasonography (POCUS), radiological imaging, and laboratory investigations are available 24 hours a day.

It was within this clinical context that the AIIMS Three-Tiered Framework was conceptualized and developed to operationalize ECC as a continuum beginning at the point of first medical contact. The framework has been implemented at our institution for more than two years and was developed as a cognitive scaffold for emergency medicine residents and physicians to facilitate the delivery of continued, definitive critical care in the ED without compromising the fundamental principles of emergency care. It also provides a structured approach to maintaining continuity of care across changes in clinical shifts. Although the three domains of ED critical care are presented as three sequential tiers for conceptual clarity, the framework is inherently dynamic and iterative, with clinicians transitioning between tiers according to the patient's evolving clinical needs. Importantly, the framework is intended to guide the care of all patients requiring ECC, irrespective of their eventual disposition. In this manner, the framework serves both as a conceptual model of ECC as a continuum and as a practical cognitive scaffold for the assessment and management of critically ill patients throughout their ED course. However, the framework is conceptual and hypothesis-generating, and its clinical impact across diverse emergency care settings requires further evaluation through prospective research.

## Conclusions

The role of the ED in the care of critically ill patients continues to evolve. As ECC emerges as a distinct domain, differing from conventional intensive care while operating within the uniquely high-stakes and dynamic environment of the ED, there is a need for structured cognitive frameworks to guide the assessment and management of critically ill patients. To address this need, the AIIMS Three-Tiered Framework for the evaluation and management of critically ill patients in the ED was conceived and implemented at AIIMS, Rishikesh.

The proposed framework conceptualizes ECC as a continuum, integrating three complementary and iterative processes: physiological stabilization, comprehensive diagnostic assessment and reasoning, and ongoing critical care management. By aligning these processes with the evolving priorities of critically ill patients in the ED, the framework provides a structured approach that may support clinical decision-making, continuity of care, education, and quality improvement. However, the framework is currently conceptual and requires further study to assess its feasibility and potential impact on patient outcomes.
